# Supplementary material for: Effect of Multimorbidity on Fragility Fractures in Community-Dwelling Older Adults: Shimane CoHRE Study
Source: J Clin Med. 2021 Jul 22;10(15):3225. doi: 10.3390/jcm10153225 (PMC8348412; doi:10.3390/jcm10153225)
Supplement: Supplementary file 1 [file jcm-10-03225-s001.zip › jcm-1259418-proofed-supplementary.pdf]

Supplementary tables

Table S1. Specific location of fragility fractures (FFx)

**Fracture site**	Total (n)	Men (n)	Women (n)
Spine	60	8	52
Sternum	2	0	2
Clavicle	4	1	3
Rib	19	1	18
Pelvis	5	2	3
Shoulder	5	2	3
Elbow	5	1	4
Carpal	7	1	6
Wrists	31	1	30
Hip/Femoral	9	1	8
Knee	5	0	5
Calf	3	1	2
Foot	34	8	26
Total	159	27	132

Note: For all subjects, men, and women, the frequency of FFx in different locations are listed separately.

Table S2. Characteristics of study participants according to falls in men and women

Variables	Men (n=556)			Women (n=864)		
	No falls	falls	*p*	No falls	falls	*p*
	n=529	n=27		n=732	n=132	
Age, years	71.5±6.4	72.7±6.9	0.13	72.3±7.0	72.8±6.8	0.47
BMI, kg/m^2	22.9±2.7	23.2±3.2	0.49	22.7±3.2	23.0±3.5	0.44
AAC Index (1-3)	167 (34.9)	23 (29.5)		235 (32.0)	39 (30.0)	
AAC Index (4-5)	193 (40.4)	29 (37.2)	0.26	288 (39.2)	49 (37.7)	0.71
AAC Index (≥6)	118 (24.7)	26 (33.3)		211 (28.7)	42 (32.3)	
HT	268 (56.1)	45 (57.7)	0.79	412 (56.1)	77 (59.2)	0.51
DM	104 (21.8)	16 (20.5)	0.80	78 (10.6)	17 (13.1)	0.41
DL	169 (35.4)	26 (33.3)	0.73	316 (43.1)	53 (40.8)	0.63
Stroke	33 (6.9)	9 (11.5)	0.15	23 (3.1)	8 (6.2)	0.09
Heart disease	71 (14.9)	16 (20.5)	0.20	76 (10.4)	15 (11.5)	0.69
Kidney disease	57 (11.9)	15 (19.2)	0.08	34 (4.6)	10 (7.7)	0.14
Anemia	40 (8.4)	8 (10.3)	0.58	156 (21.3)	37 (28.5)	0.07
Insomnia	263 (55.0)	43 (55.1)	0.99	465 (63.4)	81 (62.3)	0.82
Hyperuricemia gout	46 (9.6)	5 (6.4)	0.36	8 (1.1)	1 (0.8)	0.74
PAD	10 (2.1)	1 (1.3)	0.63	2 (0.3)	1 (0.8)	0.38
Liver disease	11 (2.3)	3 (3.8)	0.42	20 (2.7)	4 (3.1)	0.82
GD	36 (7.5)	9 (11.5)	0.23	53 (7.2)	13 (10.0)	0.27
Endocrine disease	6 (1.3)	1 (1.3)	1.00	44 (6.0)	8 (6.2)	0.94
RA	67 (14.0)	16 (20.5)	0.14	245 (33.4)	56 (43.1)	0.03
Cancer	41 (8.6)	12 (15.4)	0.06	35 (4.8)	10 (7.7)	0.17
Allergic disease	20 (4.2)	2 (2.6)	0.50	38 (5.2)	9 (6.9)	0.42
Smoke	350 (73.2)	62 (79.5)	0.24	37 (5.0)	1 (0.8)	0.03
Alcohol consumption	321(67.2)	56 (71.8)	0.42	176 (24.0)	41 (31.5)	0.07
Normal menopause	-	-	-	644 (87.7)	112 (86.2)	
Early menopause	-	-	-	60 (8.2)	14 (10.8)	0.55
Premature menopause	-	-	-	30 (4.1)	4 (3.1)	
Cognitive impairment	71 (14.9)	18 (23.1)	0.07	95 (12.9)	16 (12.3)	0.84
Number of teeth (<20)	193 (42.4)	33 (44.6)	0.73	323 (46.7)	77 (62.6)	<0.01
Denture use (yes)	251 (55.3)	39 (52.7)	0.68	399 (57.7)	83 (67.5)	0.04
Reduced maximum grip strength *	17 (3.6)	2 (2.6)	0.91	51 (7.0)	14 (10.9)	0.13
Reduced usual walking speed **	386 (81.8)	63 (81.8)	0.99	573 (79.3)	93 (73.2)	0.13
Lumbago	190 (39.7)	35 (44.9)	0.39	327 (44.6)	61 (46.9)	0.62
Parent's hip fracture history	64 (13.4)	12 (15.4)	0.63	90 (12.3)	12 (9.2)	0.32
Medication for HT	199 (41.2)	40 (51.3)	0.11	309 (42.1)	61 (46.9)	0.31
Medication for DM	87 (18.2)	14 (17.9)	0.96	64 (8.7)	16 (12.3)	0.19
Medication for DL	109 (22.8)	17 (21.8)	0.84	258 (35.1)	50 (38.5)	0.47

Note: Statistical analysis was conducted with the paired *t*-test and chi-square test, where $p<0.05$ was considered as significant.

*Reduced maximal grip strength: <28kg in men and <18kg in women, **Reduced usual walking speed by 10-m walk: ≥4.5sec.

Abbreviations: SD, standard deviation; BMI, body mass index; AAC, Age-Adjusted Charlson Comorbidity Score; HT, hypertension; DM, diabetes mellitus; DL, dyslipidemia; PAD, peripheral artery disease; GD, gastrointestinal disorders; RA, rheumatoid arthritis. Data are presented here as mean ± standard deviation. Data are also presented as number with percentage.

Table S3. Odds ratios for falls in men and women by binary logistic regression

	Men (n=556)		Women (n=864)	
Variables	OR (95% CI)	*p*	OR (95%CI)	*p*
Age	1.05 (1.00–1.11)	0.07	0.97 (0.93-1.01)	0.16
BMI	1.40 (0.38–5.22)	0.62	0.49 (0.23-1.06)	0.07
AAC Index (1-3)	-	0.26	-	0.70
AAC Index (4-5)	1.10 (0.61-1.98)	0.74	1.02 (0.48-1.61)	0.93
AAC Index (≥6)	1.61 (0.88-2.96)	0.13	1.20 (0.75-1.93)	0.44
HT	0.73 (0.37-1.43)	0.36	1.04 (0.62-1.74)	0.89
DM	1.25 (0.48-3.22)	0.65	1.23 (0.52-2.93)	0.64
DL	0.91 (0.48-1.75)	0.78	0.91 (0.56-1.48)	0.71
Stroke	1.77 (0.70-4.49)	0.23	1.75 (0.65-4.70)	0.27
Heart disease	1.28 (0.61-2.72)	0.51	1.09 (0.55-2.13)	0.81
Kidney failure	1.80 (0.85-3.84)	0.13	1.82 (0.76-4.35)	0.18
Anemia	1.22 (0.49-3.01)	0.67	1.19 (0.73-1.94)	0.49
Insomnia	0.82 (0.46-1.47)	0.51	0.72 (0.44-1.17)	0.18
Hyperuricemia gout	0.54 (0.17-1.67)	0.28	0.49 (0.04-5.55)	0.56
PAD	0.50 (0.05-4.59)	0.54	5.55 (0.34-8.94)	0.23
Liver disease	3.74 (0.78-18.08)	0.10	0.92 (0.26-3.24)	0.90
GD	1.27 (0.50-3.23)	0.61	1.37 (0.65-2.87)	0.41
Endocrine disease	1.58 (0.16-15.99)	0.70	0.93 (0.38-2.30)	0.88
RA	1.37 (0.67-2.77)	0.39	1.23 (0.78-1.96)	0.38
Cancer	1.71 (0.77-3.82)	0.19	1.80 (0.76-4.28)	0.18
Allergic disease	0.38 (0.08–1.85)	0.23	1.18 (0.48-2.93)	0.72
Smoke	1.36 (0.71-2.61)	0.35	0.14 (0.02-1.09)	0.06
Alcohol consumption	1.46 (0.76-2.80)	0.26	1.56 (0.97-2.50)	0.07
Early menopause	-	-	1.00 (0.46-2.22)	0.99
Premature menopause	-	-	0.90 (0.29-2.87)	0.86
Cognitive impairment	2.04 (1.00-4.14)	0.05	0.92 (0.48-1.78)	0.81
Number of teeth (<20)	1.20 (0.60-2.42)	0.61	2.10 (1.12-3.93)	0.02
Denture use (yes)	0.74 (0.38-1.45)	0.38	0.93 (0.50-1.71)	0.81
Reduced maximum grip strength	0.34 (0.04-3.01)	0.34	1.54 (0.72-3.28)	0.27
Reduced usual walking speed	0.94 (0.45-1.94)	0.86	0.91 (0.51-1.61)	0.74
Lumbago	1.08 (0.62-1.89)	0.79	1.01 (0.65-1.56)	0.98
Parent's hip fracture history	1.19 (0.60-2.32)	0.62	0.72 (0.38-1.36)	0.32
Medication for HT	1.52 (0.93-2.48)	010	1.16 (0.79-1.71)	0.46
Medication for DM	0.91 (0.48-1.73)	0.77	1.39 (0.76-2.52)	0.29
Medication for DL	0.89 (0.49-1.61)	0.70	1.07 (0.72-1.59)	0.74

Note: Statistical analysis was done with binary logistic regression, where $p<0.05$ was considered to be significant.

Abbreviations: OR, odds ratios; CI, confidence interval BMI, body mass index; AAC, Age-Adjusted Charlson Comorbidi Score; HT, hypertension; DM, diabetes mellitus; DL, PAD, peripheral artery disease; GD, gastrointestinal disorders; RA, rheumatoid arthritis; DL, dyslipidemia.

Table S4. Adjusted odds ratio for falls

Variables	Adjusted OR (95% CI)	*p*-value
AAC Index (1-3)	1	-
AAC Index (4-5)	1.05 (0.73-1.50)	0.79
AAC Index (≥6)	1.34 (0.92-1.94)	0.13
Men	1	
Women	1.08 (0.79-1.46)	0.64
BMI	1.04 (0.98-1.09)	0.17
Smoke	0.84 (0.51-1.38)	0.48
Alcohol consumption	1.61 (1.13-2.30)	0.01
Cognitive impairment	1.17 (0.75-1.81)	0.50
Number of teeth (<20)	1.65 (1.08-2.52)	0.02
Denture use (yes)	0.86 (0.56-1.30)	0.47
Reduced maximum grip strength	1.20 (0.64-2.24)	0.57
Reduced usual walking speed	0.93 (0.62-1.39)	0.72
Lumbago	1.06 (0.77-1.45)	0.71
Parent's hip fracture history	0.93 (0.58-1.50)	0.76
Medication for HT	1.29 (0.95-1.75)	0.10
Medication for DM	1.11 (0.72-1.71)	0.64
Medication for DL	1.02 (0.74-1.42)	0.89

Note: Falls risk was adjusted with sex, BMI, smoking, alcohol, cognitive impairment, number of teeth, denture use, reduced maximum grip strength, reduced usual walk speed, lumbago, parent's hip fracture history, medication for HT, medication for DM, and medication for DL. Statistical analysis was done with binary logistic regression, where $p < 0.05$ was considered as significant. Abbreviation: AAC, Age-Adjusted Charlson Comorbidity Score; FFx, fragility fracture; Adjusted OR, adjusted odds ratio; CI, Confidence interval; HT, hypertension; DM, diabetes mellitus; DL, Dyslipidemia.
